# Enhancing specimen collection skills for dried blood spots through an immersive virtual learning environment: a cross-sectional study

**DOI:** 10.1186/s13104-023-06584-9

**Published:** 2024-01-04

**Authors:** Hafsa Majid, Lena Jafri, Shanzay Rehman, Azeema Jamil, Fatima Khanam, Nadir Shah, Nasir Ali Khan, Aysha Habib Khan

**Affiliations:** 1 Department of Pathology and Laboratory Medicine, Aga Khan University, Karachi, Pakistan; 2 Secondary Hospitals, Aga Khan University, Karachi, Pakistan; 3 IT Academics and Computing, Aga Khan University, Karachi, Pakistan

**Keywords:** Dried blood spot, Virtual learning environment, Training, Allied health professionals, Nurses

## Abstract

**Objective:**

The quality of dried blood spot (DBS) specimens impacts newborn screening (NBS) results, hence proper training is crucial for DBS specimen collection. To address this, a training module for Allied Health Professionals (AHPs) and nurses was created on Moodle, a virtual learning environment (VLE). The purpose of this research was to determine the feasibility and effectiveness of this module.

**Methodology:**

Participants were trained on-site (March to December 2019), through online training sessions (January to June 2020), and the two training strategies were compared. Data analysis included the total number of participants, cost-effectiveness, trainer engagement, and the number of unacceptable samples collected by nurses/AHPs trained by the two strategies.

**Results:**

A total of 55 nurses/AHPs were trained on-site, while 79 nurses/AHPs completed the online module and received certificates through online VLE-based training. The trainer engagement and cost were more for onsite training. After online training, the specimen rejection rate was reduced from 0.84% (44 rejected out of 5220 total specimens collected) to 0.38% (15/3920).

**Conclusions:**

This study shows that using VLE-based DBS specimen collection training is feasible and effective for training nurses and AHPs.

**Supplementary Information:**

The online version contains supplementary material available at 10.1186/s13104-023-06584-9.

## Introduction

The goal of newborn screening (NBS) is to identify infants with treatable disorders whose symptoms and signs are not present at birth [1]. The preferred sample for NBS testing is a dried blood spot (DBS) specimen because it is simple to collect, less traumatic for newborns, has less onerous storage and transport requirements, and can be used for multiple tests from a single DBS card [2]. However, because the target analyte concentration in DBS specimens is very low, it is necessary to use assays with very low analytical sensitivity, and a high-quality DBS specimen is required [3, 4].

For the NBS test, a DBS sample is collected from heel prick by nurses or allied health professionals (AHPs) in inpatient settings [5]. Improper collection, specimen contamination, incomplete drying, and transporting at an inappropriate temperature can all degrade the quality of the DBS specimen [6, 7]. As a result, these nurses or AHPs must understand ‘what constitutes a good quality specimen’ as well as ‘how to collect, store, and transport a DBS specimen’. So that DBS specimen quality issues do not impact NBS results, because specimen rejection causes delayed reporting affecting the overall NBS program performance [8].

Nurses and AHPs are typically trained on-site for DBS sample collection and evaluated for competence. These nurses/AHPs then collect DBS specimens and transport them to the clinical laboratory, where the quality of the specimen is evaluated by a trained technologist upon receipt at the clinical laboratory for analysis [8]. However, these on-site training sessions, and competence assessments, take up a significant amount of faculty time and must be repeated for recruits or retraining of the already existing pool of nurses/AHPs. Moreover, only a restricted number of trainees can be accommodated in a single session.

In this era of technological advancements, virtual learning environments (VLEs) are becoming increasingly popular for teaching and training, offering innovative educational tools and platforms [9]. These asynchronous teaching strategies allow trainees to fulfill learning objectives at their own pace and time. A training course was created on ‘Moodle’ a VLE, to train nurses/AHPs for DBS specimen collection. This study aimed to determine the acceptability and feasibility of the VLE as a training tool for DBS specimen collection.

## Main text

### Methods

This cross-sectional study was conducted at the Biochemical Genetics Laboratory (BGL) in the Department of Pathology and Laboratory Medicine at Aga Khan University. The project team consisted of a Chemical Pathologist, a BGL supervisor, an eLearning developer, and a nurse manager. Ethical approval for the study was obtained from the institute’s ethical review committee (ID # 2022-7346-20976). All participants gave written consent by electronic signature to participate in the study.

The study was conducted in four hospitals affiliated with Aga Khan University, including Stadium Road Hospital (a tertiary care center) and three maternal and child hospitals, Karimabad Hospital, Garden Hospital, and Kharadar Hospital. At these hospitals, full-term newborn babies were admitted to the well-baby nursery of their respective hospitals, while premature and critically ill newborns, were admitted to the Neonatal Intensive Care Unit (NICU), and DBS specimens were collected for NBS by nurses/AHPs. These nurses and AHPs were trained for DBS specimen collection, where the AHPs included phlebotomists and NICU technicians.

The onsite training was provided to nurses and AHPs specifically at the Stadium Road Hospital, and their competence was assessed afterward. Alternatively, an online training course was developed on “Moodle,“ a VLE, nurses, and AHPs from all three hospitals were trained through this online platform, followed by an assessment. The key performance indicators (KPIs) for the two training methods, including the number of unacceptable samples, were then compared. The clinical laboratory was responsible for receiving and analyzing all DBS specimens to monitor this KPI.

### Onsite training for the DBS specimen collection

**Fig. 1:**
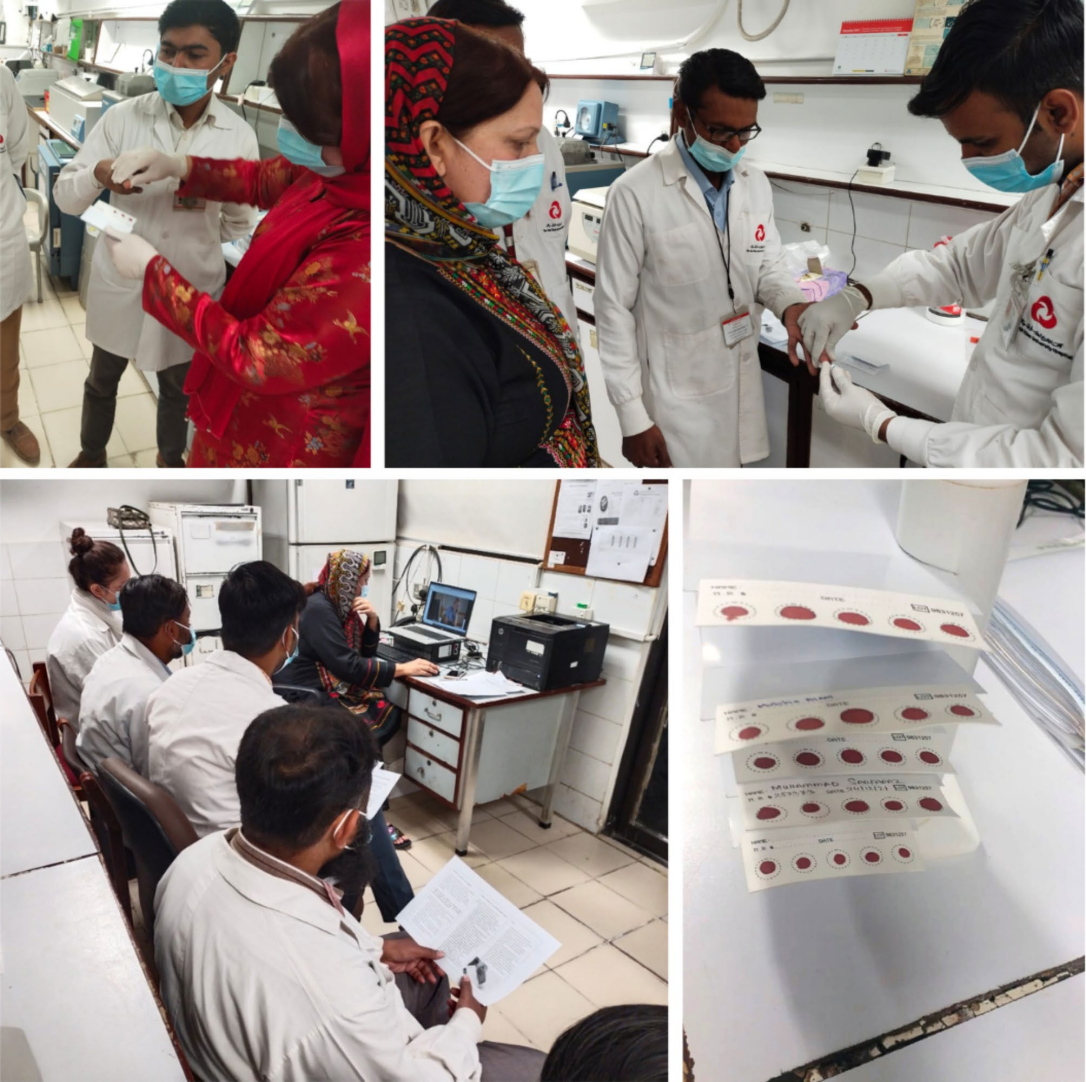
Onsite training of allied health professionals for dried blood spot collection

Only nurses and AHPs at the Stadium Road Hospital received physical or on-site training. The trainers for these sessions were an NBS expert and a BGL scientist, and a total of three sessions were conducted (Fig. 1). Each session lasted half a day and included instructional lectures/videos on DBS specimen collection, an overview of the institute’s current NBS program, relevant policies, and diagnostic protocols for the disorders, followed by a discussion and a question-answer session. Additionally, the training involved a demonstration by expert trainers on the proper collection, drying, and transportation of DBS specimens. Following the demonstration, the nurses’/AHPs’ performance was directly observed, and their competence in the procedure was assessed.

### Online module development on VLE for DBS specimen collection

An online course was created on Moodle for DBS specimen collection training. The Clinical and Laboratory Standards Institute (CLSI) NBS01-A6 guidelines and ‘Laboratory General,‘ and ‘Clinical Biochemical Genetics’ Checklists of the College of American Pathologist Standards were used to create all content [10, 11]. The group developed a contextual curriculum that included instructional materials such as videos, a presentation on the NBS program at the Aga Khan University, and a DBS manual including specimen collection site, procedure, specimen handling, characteristics of good and bad quality specimens, frequency of training and criteria for retraining. The DBS manual also included contact information for relevant individuals, such as well-baby nursery nursing managers, neonatal intensive care units, BGL technologists, Paediatric endocrine and metabolic team representatives, and NBS experts.

For knowledge assessment, a quiz was created, and competency assessment forms were uploaded to Moodle. After a trainee received 100% on the quiz and passed the competence assessment, the certificate was generated. To ensure content validity and reliability of instructional materials, two non-team members piloted the module to evaluate both the content and the course. The objective was to assess the difficulty, reliability, and accuracy of both the quizzes and study material, ensuring their suitability before implementing the module.

### VLE-based training for DBS specimen collection

This module was implemented from January to June 2020, and nurses were enrolled to participate in the training module. The train-the-trainer approach was employed for this module, where four trainers were carefully selected to perform a competence assessment. Before introducing the module to the participants, these trainers read the material, and completed quizzes, after direct observation of DBS specimen collection on a newborn baby, the BGL scientist assessed their competence. These trainers then enrolled and trained other nurses in their respective areas for DBS specimen collection on Moodle. The decision to use the train-the-trainer approach was motivated by the large number of learners involved. Scalability, efficiency, and uniformity in the training process were attained by teaching a small number of trainers who could then assess the competence of a broader audience.

### Statistical analysis

Analysis was performed using Excel 2020. The feasibility was evaluated based on the number of nurses/AHPs trained; the cost of online/onsite courses; the time of trainer for developing content, implementing the online course, or conducting onsite sessions, and the ratio of nurses/AHPs trained to trainer time. To assess the effectiveness of training, unacceptable DBS specimens received by the nurses/AHPs trained by the two strategies were compared. A specimen was labeled as unacceptable if any of the following criteria was met, incomplete drying, in sufficient quantity, clotting or layering, presence of serum rings, or contamination.

## Results

During the three on-site sessions, 53 nurses and 2 AHPs from the well-baby nursery and NICU underwent training, and their competence was evaluated by trainers. Over the following twelve months, the nurses and AHPs were monitored to assess the occurrence of unacceptable specimens. Table 1 provides information on the associated costs, trainer engagement, and the percentage of unacceptable specimens collected by nurses/AHPs trained via onsite sessions.

In Jan 2021, an online training module was developed on Moodle. The trainers included NBS experts (n = 4), and BGL scientists (n = 2). Over a period of six months, 120 nurses/AHPs were enrolled in the online training module (supplemental Fig. 1). Among the participants, there were 106 nurses from four different hospitals [Stadium Road Hospital (n = 3), Karimabad (n = 26), Garden (n = 39), and Kharadar (n = 38)]. While 14 AHPs, including NICU technicians and phlebotomists, were also enrolled. The participants attempted the knowledge assessment quizzes, a total of 234 times with 222 completed attempts. Out of the total, only, 104 scored 100%, 48% (n = 50) attempted the quiz once, 23% (n = 24) twice, 13.5% (n = 14) three times, and 15.4% (n = 16) more than three times. The quiz took approximately 7.2 min (4–60) to complete, and the median score was 17.3 (18 − 2.4). The total number of quiz attempts showed a positive correlation with the quiz score (r 0.33, p-value 0.001), but an inverse correlation with the total number of attempts (r -0.13, p-value 0.049). After achieving a perfect score, 79 nurses or AHPs (75.9%) cleared on competence assessment and received training certificates. The KPI was monitored for 6 months after the online training via Moodle. Table 1 provides information on the associated costs, trainer engagement, and the percentage of unacceptable specimens collected by nurses/AHPs trained via onsite and online sessions.

**Table 1:** Comparative analysis of onsite and online training: costs, trainer engagement, and specimen quality

Feasibility Parameter	Definition	On-site Training (Jan-Dec 2020)	VLE Training (Jan-Jun 2021)
**Accessibility and learner engagement**	Number of learners trained	55	104
**Cost-effectiveness**	Cost of training	175 USD	375 USD
**Instructor engagement**	Instructors’ time engagement	24 h	30 h
	The ratio of the number trained and hours of engagement	2.2916	3.4667
**KOI for the Unacceptable Sample**	Samples received	5220	3920
	DBS not soaked properly	29	5
	Less than 2 complete circles in a filter	9	6
	Sample quantity not sufficient for analysis	5	3
	Clotted sample	1	1
	Serum rings	0	0
	The sample was not dried properly	0	0
	Contaminated sample	0	0
	Total unacceptable samples	44 (0.84%)	15 (0.38%)

The KPI improved after VLE-based training, Table 1. However, the report comparing the two strategies for this KPI was not statistically significant (p-value 0.3384). Table 2 presents a detailed comparison of the two training methods for DBS specimen collection.

**Table 2:** Comparison of two training methods for DBS Specimen collection training

Onsite Training	VLE based Trainings
Faculty time is required for both developing and delivering the course	One-time faculty engagement is required in developing the course and at the time of competence assessment. However, it imposes less burden on faculty time compared to onsite training.
Synchronous sessions, hence, not all participants can attend them at the same time	Asynchronous teaching-AHPs can attempt the course at their own pace and time.
Need to be arranged repeatedly for the new AHP recruits	Once developed, the course can also be used to train new AHP recruits.
Separate sessions need to be conducted for hospitals in different areas. And not many AHPs can be trained at one time.	Multiple AHPs of different areas, /hospitals can be trained at the same time.
Need larger spaces/conference rooms to conduct sessions, to abide by the SOPs post-COVID	A mobile application for Moodle can be made available to AHPs.
Logistic and administrative support required	Minimal logistics are required for the competence assessment only.
Competence assessments can be done onsite	Competence assessments cannot be done. You need to call AHPs onsite for it.
Require laptop, multimedia, and appropriate conference room/space to conduct onsite sessions	Require licensed VLE, training of persons to develop and conduct courses via VLEs

## Discussion

This study encompassed both AHPs and Nurses since both play a crucial role in collecting dried blood spot specimens. In our clinical setup, AHPs primarily included phlebotomists and NICU technicians. Although nursing is not typically classified as an AHP according to the Association of Schools of Allied Health Professionals [12], we included both nurses and AHPs in this study due to their involvement in DBS specimen collection. On-site training posed challenges in terms of resource allocation and time commitment from trainers. To address these issues, an online course was developed as a solution to overcome these limitations and facilitate more accessible training methods.

According to our study findings, the VLE-based platform was feasible and effective in training nurses or AHPs for DBS specimen collection. The online course was more cost-effective, had improved accessibility, flexibility for trainees to access at their own pace, scalability with minimal infrastructure cost, and training a large number of participants with minimal trainer engagement. Another factor could be the availability of downloaded educational resources during the online modules, allowing AHPs to reference them as needed. However, certain limitations were no face-to-face interaction with the trainers and technological requirements, such as good Wi-Fi connectivity, and devices that can support VLE platforms. Similar findings were reported in a study by Al Rawashdeh AZ et al., which reported that online learning strategies improve accessibility and flexibility but decreases teacher-student interaction, leading to social isolation [13].

The quality indicator for the unacceptable DBS specimen improved after the online training module was implemented, with the percentage of unacceptable specimens decreasing by more than half, from 0.84 to 0.38%. This could be because we were able to train a greater number of trainees with minimal logistic requirements and trainer engagement using the VLE-based training. Similar findings were reported by MacRae D JM et al. that online training is flexible, scalable, can train a large number of trainees, and trainees can complete these courses at their own pace. The participants have complete control over their learning and can go back and read up on areas where they believe they are lacking, since the course content can be accessed at any point in time. Moreover, they can devote more time to a specific topic while skipping over irrelevant content [14].

According to a study by Veenhof H et al., the utilization of a web-based application for training in DBS specimen collection resulted in a decrease in the number of unacceptable specimens and an increase in the percentage of satisfactory specimens to 95.9%. The purpose of this application was to provide an objective evaluation of the quality of DBS specimens [15]. The current study also demonstrated similar findings, with a reduction of over 70% in the proportion of unacceptable specimens. Another study conducted by Allen MA et al. reported similar findings, they evaluated the acceptability and feasibility of web-based multimedia training for DBS specimen collection. Their findings showed that 96% of the collected specimens were of good quality and usable. As a result, they concluded that this training method is viable for instructing non-healthcare providers in the self-collection of specimens [16].

DBS specimens are not only utilized for routine patient testing but are also gaining popularity in research applications. The primary advantage of DBS specimen collection is its user-friendly nature, allowing it to be conducted outside of hospital settings by non-healthcare personnel. They are being used for various purposes such as drug analysis, toxicology studies, viral load testing, and routine chemistry analyses [17, 18]. Currently, many NBS programs are preserving surplus DBS samples for future utilization in research, including method validation for new biomarkers or conducting repeat analyses and testing [19]. However, the accuracy of the results is directly associated with the quality of the DBS specimen. Therefore, proper training of nurses and AHPs becomes critical for obtaining good-quality DBS specimens [20]. The study’s findings suggest the use of VLE-based training for DBS specimen collecting to promote standardized and high-quality specimen collection. Online training modules on VLE systems provide a practical and efficient alternative to training large groups of learners and can be more cost-effective than on-site training sessions when considerations such as trainer involvement and associated costs are considered.

## Conclusion

The study’s findings highlight the effectiveness of VLE-based platforms in delivering DBS specimen collection training to nurses/AHPs, which is crucial for ensuring the availability of high-quality specimens for accurate specimen collection. VLE-based training offers scalability and flexibility, enabling the training of a larger number of students while minimizing logistical constraints and trainer involvement.

### Limitations

Limitations of this study include the relatively small sample size, as only 55 nurses/AHPs were trained on-site and 79 completed the online module. The study duration of March 2019 to June 2020 might not capture long-term effects or variations over time. Also, the curriculum developed was contextual so the study may not be generalizable. Hence, studies with larger sample sizes, longer duration, and diverse settings are needed to validate the findings and address these limitations.

### Electronic supplementary material

Below is the link to the electronic supplementary material.

Supplementary Material 1

## Data Availability

The data set used and analyzed in the current study is available from the corresponding author upon reasonable request. Temporary access to the training module on Moodle (https://vle.aku.edu/course/view.php?id=2637) can be provided on request.

## List of abbreviations

VLE: Virtual learning environment
DBS: dried blood spot
BGL: biochemical genetics laboratory
NICU: neonatal intensive care unit
CLSI: Clinical laboratory standards institute
KPI: key performance indicator
