# Supplementary material for: Enhancing specimen collection skills for dried blood spots through an immersive virtual learning environment: a cross-sectional study
Source: BMC Res Notes. 2024 Jan 4;17:16. doi: 10.1186/s13104-023-06584-9 (PMC10768425; doi:10.1186/s13104-023-06584-9)
Supplement: Supplementary file 1 — Supplementary Material 1 [file 13104_2023_6584_MOESM1_ESM.docx]

**Supplemental Figure 1: Nurses and AHPs trained using the online VLE.**
